# Characterizations of Rapid Sintered Nanosilver Joint for Attaching Power Chips

**DOI:** 10.3390/ma9070564

**Published:** 2016-07-12

**Authors:** Shuang-Tao Feng, Yun-Hui Mei, Gang Chen, Xin Li, Guo-Quan Lu

**Affiliations:** 1 Key Laboratory of Advanced Ceramics and Machining Technology of Ministry of Education, Tianjin University, 135# Yaguan Road, Jinnan District, Tianjin 300350, China; 13207567335@163.com (S.-T.F.); xinli@tju.edu.cn (X.L.); gqlu@vt.edu (G.-Q.L.); 2 Tianjin Key Laboratory of Advanced Joining Technology and School of Materials Science and Engineering, Tianjin University, Tianjin 300350, China; 3 School of Chemical Engineering and Technology, Tianjin University, Tianjin 300350, China; agang@tju.edu.cn; 4 Department of Materials Science and Engineering, Virginia Tech, Blacksburg, VA 24061, USA

**Keywords:** nanosilver, die-attach, current-assisted sintering, rapid joining, characterization

## Abstract

Sintering of nanosilver paste has been extensively studied as a lead-free die-attach solution for bonding semiconductor power chips, such as the power insulated gated bipolar transistor (IGBT). However, for the traditional method of bonding IGBT chips, an external pressure of a few MPa is reported necessary for the sintering time of ~1 h. In order to shorten the processing duration time, we developed a rapid way to sinter nanosilver paste for bonding IGBT chips in less than 5 min using pulsed current. In this way, we firstly dried as-printed paste at about 100 °C to get rid of many volatile solvents because they may result in defects or voids during the out-gassing from the paste. Then, the pre-dried paste was further heated by pulse current ranging from 1.2 kA to 2.4 kA for several seconds. The whole procedure was less than 3 min and did not require any gas protection. We could obtain robust sintered joint with shear strength of 30–35 MPa for bonding 1200-V, 25-A IGBT and superior thermal properties. Static and dynamic electrical performance of the as-bonded IGBT assemblies was also characterized to verify the feasibility of this rapid sintering method. The results indicate that the electrical performance is comparable or even partially better than that of commercial IGBT modules. The microstructure evolution of the rapid sintered joints was also studied by scanning electron microscopy (SEM). This work may benefit the wide usage of nanosilver paste for rapid bonding IGBT chips in the future.

## 1. Introduction

Insulated gated bipolar transistors (IGBTs) have become important Device for power systems applications such as high-voltage direct current transmission, lamp circuit and variable speed drives, and traction [1,2]. The requirements in size, weight, reliability, durability, ambient temperature, and environment are driving the operation temperatures of power electronics higher than 200 °C [3]. It is known that materials and packaging technologies play more and more important roles in the field of power electronic packaging. In order to reduce the junction temperature of silicon-based IGBT modules, more and more attention has been paid to packaging technologies and materials [4].

In order to avoid the effects of lead, people proposed many lead-free solders, i.e., Ag-Sn or Au-Sn solder [5,6]. Although these modules can work at the high temperature of 200 °C, the short lifetime or high cost still restrict their application. More and more attention has been paid to using nanosilver paste in power electronic industry, especially for high temperature applications [7,8,9,10]. Compared with traditional lead-tin and lead-free solder, which are widely used as the die-attach materials for power electronics, low-temperature sintering of nanosilver paste has become a promising lead-free chip joining method. This is attributed to its superior thermal conductivity, electrical conductivity, and reliability because of its high melting point (960 °C) [11,12]. Bai et al. [13] have demonstrated that the fabrication of high temperature devices using nanosilver paste presented superior characteristics over solder joints, including better electrical, thermal and mechanical properties. Ogura et al. [14] found that diode packages made with sintered silver interconnects had electrical and thermal properties equal to those with lead-soldered interconnects.

However, the conventional way to sinter nanosilver requires either a relatively long processing time (up to one hour) under zero pressure or hot pressing, during which the parts are under uniaxial stresses of several megapascals for tens of seconds to a few minutes at the sintering temperature [15,16,17]. Moreover, this time-consuming process may also cause excessive grain growth [18], which may decrease the mechanical properties of sintered nanosilver and then limit its applications.

A number of novel sintering methods, such as microwave sintering [19], selective laser sintering [20], and electric-current-assisted sintering [21,22,23,24], have been put forward to improve the efficiency and properties of sintered materials. Microwave sintering [19] and selective laser sintering [20] are usually used to sinter metals/metal matrix composites and ceramics, while electric-current-assisted sintering can be used for joining materials with high mechanical performance [23]. Recently, a concept of rapid sintering of nanosilver paste using pulsed current for joining, e.g., bus-bar interconnection, has been studied [23]. It has received attention in recent years because of many advantages: extremely high heating rate, 100~1300 °C/s, which could help to bypass the low temperature regime and avoid the aggregation of the nanoparticles; short sintering time, which is a benefit for improving the efficiency; and almost no grain growth, which could lead to the enhancement of mechanical properties of sintered nanosilver. Allen et al. [25] used electrical current assisted sintering (ECAS) to sinter nanosilver on temperature-sensitive photopaper. The conductivity of the sintered nanosilver reached as high as 3.7 × 10^7^ S·m^−1^. Mei et al. [23] used ECAS to bond copper plates by sintering of nanosilver in less than one second and the sintered joints show high shear strength, i.e., 40 MPa. Extremely high heating rates also make it possible to sinter nanoparticles with insignificant grain growth [26], leading to fine grains, i.e., 300 nm on average, and superior mechanical properties.

Although we had bonded copper plates successfully using the alternative current (AC) by sintering of nanosilver paste before [23] and the properties of the sintered nanosilver by electrical current are good, we only used it for bonding copper plates as bus-bar interconnection. Unfortunately, combination of AC of more than 6.0 kA and pressure of more than 10 MPa had to be used to get a robust sintered nanosilver joint in our previous work. It is doubted whether the method could be used for attaching power chip because the semiconductor chips are not conductive and could not take such high current. The objective is to bond IGBT chips with a substrate by sintering nanosilver paste using electrical current and characterize its mechanical, thermal, microstructural, and electrical properties. In this paper, we have bonded IGBT chips successfully with copper plates electroplated with silver rapidly by sintering of nanosilver paste at low current, i.e., 2.0 kA, and low pressure i.e., 1 MPa. This rapid joining method can be used to form robust die attachment by avoiding excessive current breakthrough power semiconductor chip.

## 2. Materials and Methods

**Figure 1:**
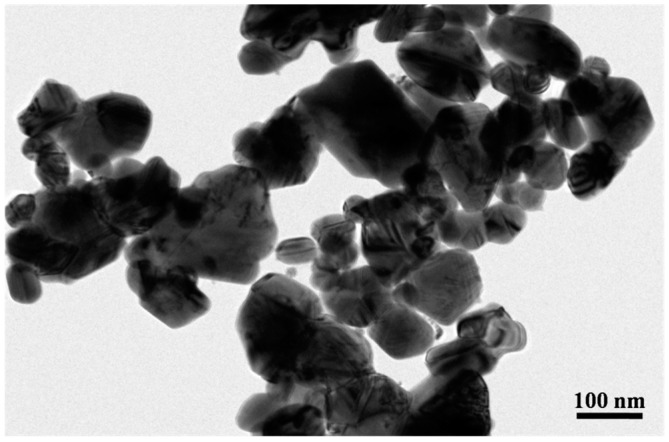
A transmission electron microscopic image of silver nanoparticles.

Figure 1 shows a TEM micrograph of silver nanoparticles in the paste used in this study. The composition of the paste is present in our previous work [23]. These nanoparticles have a wide particle size distribution from 20 to 140 nm. The average size is 50 nm. The nanosilver paste was prepared by mixing selected organics, surfactants, and binders with silver nanoparticles. The organics can prevent aggregation or agglomeration of the silver nanoparticles at low temperatures, e.g., below 200 °C. Once the temperature is increased higher, most of the organics will be burned out. Then, the silver nanoparticles can experience favorable densification by grain boundary diffusion [27]. Evident silver-silver necks could be formed among these particles uniformly.

**Figure 2:**
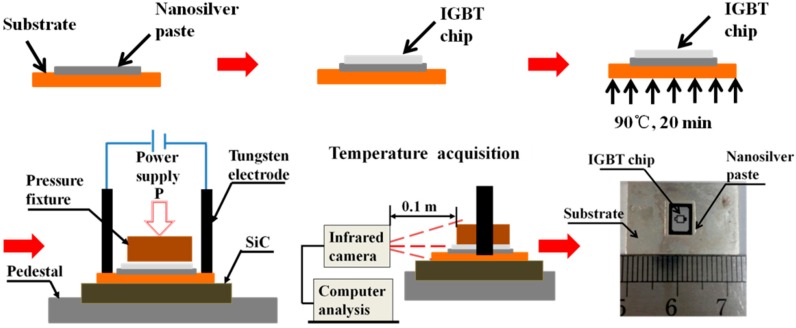
Schematic diagram of pulse-current-assisted sintering process.

The substrate used for attaching IGBT chips is made of copper with 5 µm silver coating on the surface. The dimension of the substrate is 23 mm × 15 mm × 1.5 mm. Before printing nanosilver paste, the substrate should be cleaned ultrasonically in alcohol for 30 min. Then a square layer of nanosilver paste (8 mm × 6 mm × 0.09 mm) was stencil-printed on the substrate. An IGBT die (6.5 mm × 4.87 mm × 0.12 mm, 1200 V, 25 A, SIGC32T120R3LE) was picked and placed on the as-printed paste. The specimens were first pre-dried on a heating plate for 0 to 30 min after slowly heating from room temperature to the pre-dried temperature at a heat rate of 5 °C/min in order to remove most of the solvents in the paste. Then the pre-dried specimen was sintered using pulsed current for several seconds, i.e., 90 s, 120 s, 150 s, and 180 s, under a low pressure. The schematic diagram of the current sintering process is shown in Figure 2. The power source is able to provide both pulsed and continuous electric current up to 10 kA. A self-design fixture was used to position the pre-dried specimen. A piece of SiC was used here as an insulation to avoid current flowing elsewhere and reduce the heat dissipation to the base. We used SiC in this work because the SiC is rigid and insulative enough with relatively low thermal conductivity. The temperature distribution of the as-printed nanosilver paste during sintering was measured by an infrared radiation camera, which was placed in front of the sintering equipment. A clear image of the temperature distribution contour of the nanosilver joints could be achieved by adjusting the focal length and the height of the tripod. A typical as-sintered specimen is also shown in Figure 2. The specimen was sintered in air under the combined condition of the sintered current of 2.0 kA, the current-on time of 150 s, and the assisted pressure was 1 MPa.

It is critical to monitor the temperature profile of the sintering process in order to get robust as-sintered nanosilver joints. However, it is inconvenient to measure the temperature profile by conventional ways, e.g., thermal couple, because the thermal couple should be mounted on the surface of region-of-interests (ROI) and could only measure the temperature locally. An infrared radiation (IR) camera (Guangzhou SAT Infrared Technology Co., Ltd., SAT G90, Guangzhou, China) was adopted to measure the temperature distribution and evolution of the rapid current-assisted sintering process. The accuracy of the results by the IR camera should be highly dependent on the determination of emissivity. As a result, the reference channel method [28] was used to obtain accurately the emissivity of the average temperature of the nanosilver layer and surrounding area (ROI). A specific thermal stable black paint (Botny, 550 °C High temperature paint, Guangzhou, China) with the constant emissivity, i.e., 0.95, was used to cover the ROI of the pre-dried specimen. It is difficult to measure the temperature profile of the nanosilver layer, because evaporation of the solvent along with burnout of most of the organics in the paste, and densification of the silver particles during the sintering process will cause shrinking of the thickness of the nanosilver layer [29].

**Figure 3:**
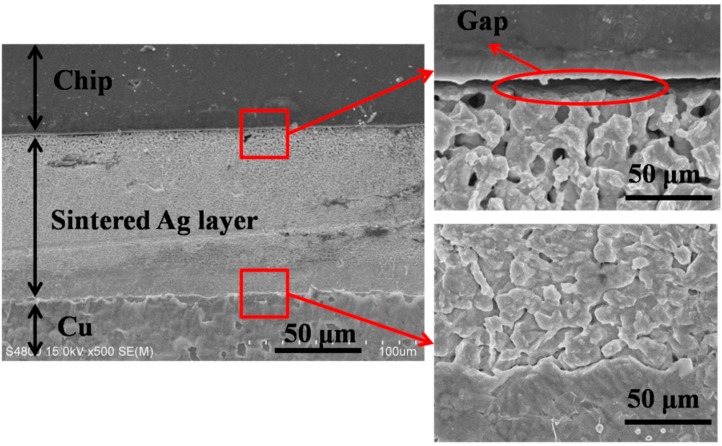
Scanning electron microscopic images of cross section of sintered silver joint without pressure.

Figure 3 shows the cross section of the sintered joint without introducing fixing pressure. There is a significant gap between the IGBT chip and the sintered nanosilver. It is likely that the rapid temperature ramping by the electrical current heating caused the abrupt outgassing of the organics in the paste. Significant force was induced by the abrupt outgassing of the organics and could drive the IGBT chip away from the paste. As a result, the gap could be generated at the interface between the IGBT chip and the paste in this case. The gap should hinder the further atomic migration of silver during sintering to generate robust joint. In order to get rid of the unexpected gap, a fixture was designed to provide ~1 MPa pressure on the chip in this work.

**Figure 4:**
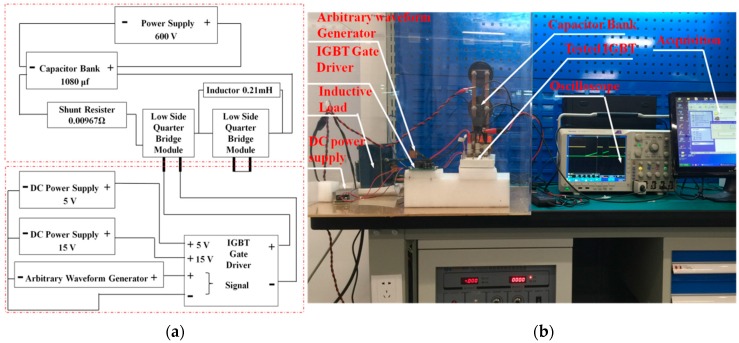
(**a**) Schematic diagram of a circuit; (**b**) a picture of electrical connects of the double pulse testing.

The electrical properties of the as-sintered IGBT assemblies, i.e., switching on/off performance, were characterized by double pulse testing [30] in order to verify the feasibility of the rapid sintering method for bonding power chips. Figure 4a shows the circuit of the double pulse testing schematically. Figure 4b show the electrical connects of the double pulse testing.

Gain/particle size of the sintered nanosilver was also measured to correlate with the sintering process. The most widely used method of average grain/particle size measurement is the mean lineal intercept. In order to prepare samples for the measurement, the fracture surface of sintered nanosilver was dipped for 4 s in an etching solution of 30 vol % ammonia (NH_4_OH), 43 vol % hydrogen peroxide (H_2_O_2_), and 27 vol % distilled water (H_2_O), and then washed by distilled water. The microstructures of etched fracture were observed by SEM to reveal the particle size. The etched fracture surface was analyzed at different regions at least three times by SEM. According to ASTM E112-96, the mean lineal intercept length is the average length of a line segment that crosses a sufficiently large number of grains. It is proportional to the equivalent diameter of a spherical grain. The mean lineal intercept length is determined by laying a number of randomly placed test lines on the image and counting the number of times that grain boundaries are intercepted. Mathematically, it is defined as:

(1) $$\bar{L_{L}}\;=\;\frac{1}{\bar{N_{L}}}\;=\;\frac{L_{T}}{\text{PM}}$$

where N_L_ is the number of intercepts per total length of the test lines L_T_; P is the total number of grain boundary intersections and M is the magnification. All grains/particles in each SEM pictures were measured with a plurality of lines to obtain the average size in this work.

A summary of sintering conditions using electrical current is listed in Table 1. At least three samples were prepared for each condition. The effects of electrical current, current-on time, pre-drying temperature, and pre-drying time on robustness of the sintered IGBT assemblies were evaluated in this work.

Thermal properties of these sintered samples were measured by thermal gravimetric (TG) in air at different final temperature and time with the heating rate of 5 °C/min.

A die-shear tester (XTZTEC Condor 150) was used to measure the shear strength of the as-sintered specimens at a displacement rate of 4 × 10^−4^ m/s. The thermal resistance of the IGBT assembly using nanosilver paste was characterized by a self-developed thermal impedance measurement system [31]. The fracture surface and the cross-section of the joint were analyzed by scanning electron microscopy (SEM).

The void ratio of the joint was measured by X-ray computed micro-tomography (μ-CT). To identify the void regions from the CT images, an appropriate threshold should be determined. Regions with pixels below this threshold are treated as voids. The details of the methods can be found in the references [32,33,34]. Since the minimum void size that can be detected by the X-ray tomography is 10 μm, we defined a void in this work as defects that are larger than 10 μm. Comparison of the microstructures and the voids of all the sintered joints were discussed to clarify the relationship among processing, performance, and microstructures.

## 3. Results

### 3.1. Temperature Profile

**Figure 5:**
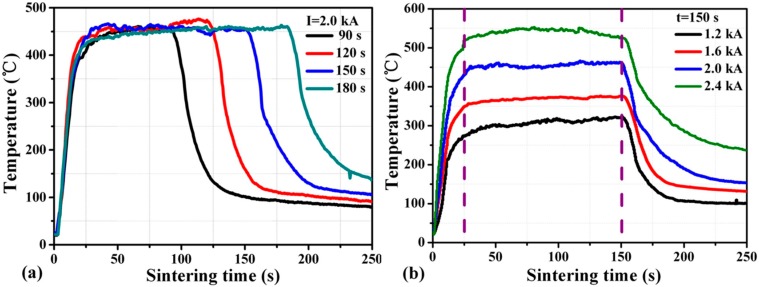
Temperature profile of nanosilver joints sintered at different: (**a**) current-on time; (**b**) sintering current.

Figure 5a shows the temperature variation of joint during the sintering process under the current of 2.0 kA with different current-on time, i.e., 90 s, 120 s, 150 s, and 180 s. The temperature of the joint rises rapidly at the beginning of the process, i.e., 25 s, with a heating rate of >20 °C/s because of massive instant Joule heat. The heating rate is constant before the peak temperature reaches ~450 °C regardless of the current-on time. Figure 5b shows that the peak temperature could reach almost 550 °C once the sintering current is 2.4 kA. It is concluded that the heating rate and the peak temperature are only dependent on the sintering current and independent of the current-on time.

### 3.2. Die-Shear Strength and Thermal Resistance

Die-shear strength is one of key factors affecting the mechanical performance and reliability of die attachment [35]. We had realized robust sintered joint for bonding copper plates, i.e., 25 mm^2^, by current-assisted sintering of nanosilver paste [7]. It is essential to achieve die-shear strength as high as the conventional solders, hot-pressing sintered nanosilver, i.e., 30 MPa. Furthermore, the thermal property of the IGBT assembly is also important to guarantee the production consistency and reliability, especially for high temperature and high power applications. An improved transient thermal impedance (Z_th_) measurement system was self-developed [31], using the electrical method with V_ge_ of the IGBT as a temperature-sensitive parameter.

**Figure 6:**
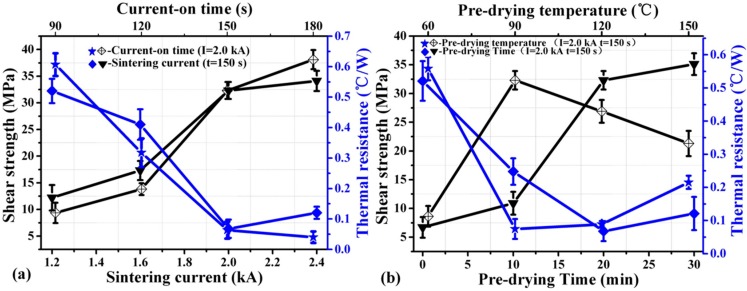
Shear strength and thermal resistance of nanosilver joints sintered: (**a**) at different current (current-on time of 150 s, pre-drying temperature of 90 °C, and pre-drying time of 20 min), at different current-on time (sintering current of 2.0 kA, pre-drying temperature of 90 °C, and pre-drying time of 20 min); (**b**) at different pre-drying temperature (current-on time of 150 s, sintering current of 2.0 kA, and pre-drying time of 20 min), at different pre-drying time (current-on time of 150 s, sintering current of 2.0 kA, and pre-drying temperature of 90 °C).

The heat of the sintering process is joule heat which is generated when current flows through the substrate, and it can be expressed as Q = I^2^Rt. The sintering current and current-on time have great influence on the temperature. Figure 6a shows that the average thermal resistance and shear strength of the IGBT assemblies is strongly dependent on the sintering current and current-on time.

The average thermal resistance of the sintered samples decreases as the current-on time increases. At the same time, the average thermal resistance decreases with increasing the sintering current from 1.2 kA to 2.0 kA. When increasing the sintering current from 2.0 kA to 2.4 kA, however, the average thermal resistance increases slightly. The shear strength of the IGBT assemblies increases as the sintering current and the current-on time increases. The die-shear strength is only ~12 MPa once the current is 1.2 kA and the current-on time is 150 s. In this case, the thermal resistance of the IGBT assembly could be as large as ~0.5 °C/W. Furthermore, the die-shear strength is less than 10 MPa, even when the current increases to 2.0 kA with the current-on time of 90 s. It is likely that the organics could not be burnt out adequately and densification of the silver particles is insufficient once the sintering current or the current-on time is at low levels [23]. If a large amount of organics remained in the paste, strong bonds could not form because the residual organics may hinder atomic inter-diffusion of the silver particles and heat conduction [36,37]. Consequently, the larger sintering current and the longer current-on time could accelerate volatilization, decomposition, or ablation of the organics [7]. Inter-granular diffusion happens between silver particles at high temperatures and the higher current magnitude accelerates the diffusion of silver atoms as well as density. A large amount of heat and local high temperature increase the rate of diffusion of silver atoms and then benefit forming a clear neck between particles, which is consistent with Akada et al. [36].

The larger sintering current also resulted in the larger heating rate, which should be beneficial to reduce non-densification diffusions, i.e., surface diffusion, by bypassing the low-temperature regime instantaneously [36]. The non-densification diffusion is able to consume the driving force for further densification diffusion because the driving force for sintering of nanosilver paste is the tendency to reduce the free energy of the Ag nanoparticles, accomplished by material transport from high energy site to lower one.

Moreover, the extremely large heating rate is prone to form a large amount of twins, which increases the thermal conductivity of the sintered nanosilver [38]. Therefore, the die-shear strength of the IGBT assembly could reach as high as ~35 MPa and the thermal resistance could reduce to only 0.06 °C/W in the condition of 2.0 kA and 150 s.

**Figure 7:**
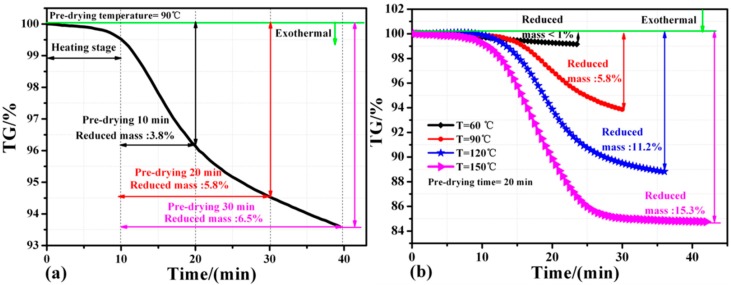
Thermal gravimetric trace of nanosilver paste: (**a**) heated to 90 °C for different durations; (**b**) heated to different temperatures for 20 min at a rate of 5 °C/min.

Figure 6b shows that the effect of pre-drying temperature and pre-drying time on the average die-shear strength and the thermal resistance of the sintered silver joints. It can be seen that the die-shear strength increases and the thermal resistance decreases with increasing pre-drying temperature from 60 °C to 90 °C. However, when increasing the pre-drying temperature from 90 °C to 150 °C, the die-shear strength decreases and the thermal resistance increases. The thermal resistance was 0.52 °C/W, 0.25 °C/W, 0.07 °C/W, and 0.12 °C/W under the pre-drying time of 0 min, 10 min, 20 min, and 30 min, respectively. The die-shear strength increases with increasing pre-drying time. It is likely that a large amount of organics was burned out once the temperature reached 90 °C or even higher because a significant weight loss at ~90 °C, as shown in Figure 7b. Therefore, the pre-dried nanosilver paste became too dry to be deformed and wet due to the burning out of the large amount of the organics when the pre-drying temperature increased from 90 °C to 150 °C. Many defects or air gaps might be present in the interface between the over-dried nanosilver paste and the power chip and hinder further atomic diffusion of silver particles that is the bonding mechanism of sintering of the nanosilver paste although the fixing pressure is helpful to reduce the air gaps at the interface to some extents. The die-shear strength and the thermal properties reduced consequently. The fixing pressure of 1 MPa is too small to avoid most of the defects and the air gaps if the pre-dried paste is too dry and not deformable. It is also not recommended to increase the fixing pressure to a much higher value, e.g., 10 MPa, because there is a risk damaging the chips under such high pressure. It is concluded that the pre-dried temperature is critical to the bonding quality of the sintered nanosilver paste using electrical current and should not be higher than ~90 °C based on the TG results.

In order to determine the appropriate pre-drying conditions, thermal properties of these sintered samples were measured by thermal gravimetric (TG) in ambient atmosphere with different pre-drying temperature and different pre-drying time at the heating rate of 5 °C/min. The results are shown in Figure 7. Figure 7a shows that with the increase of pre-drying time the more mass loses of the paste. The mass reduction of the paste with a pre-drying time of 30 min is only 0.7 wt % more than that of the paste with a pre-drying time of 20 min at 90 °C. It indicates that there is no need to prolong the pre-drying time to more than 20 min. The conclusion is also supported by the results of die-shear strength and thermal resistance, as shown in Figure 6b.

Figure 7b shows that the mass reduction of the paste is less than 1%, ~5.8%, ~11.2%, and ~15.3% when pre-drying at 60 °C, 90 °C, 120 °C, and 150 °C for 20 min, respectively. It should be noted that it takes several minutes to heat the paste to the pre-dried temperatures before the temperature remains constant for 20 min. For example, in the case of 90 °C for 20 min as shown in the red line of Figure 7b, it takes 13 min to heat the paste from ambient temperature to 90 °C. As a result, the process lasts 33 min in total. It is also worth noting that the mass reduction of the paste increases to 11.2 wt % and 15.3 wt % once the pre-dried temperature increases to 120 °C and 150 °C, respectively. Such great reduction was due to the evaporation of more organics in the paste and likely led to the defects or delamination at the interfaces among the IGBT, the paste, and the substrate. It is suggested that the evaporation of the organics should be controlled as ~6 wt % in order to improve the joint quality. The conclusion is also supported by the results of die-shear strength and thermal resistance, as shown in Figure 6b.

### 3.3. Electrical Properties

**Figure 8:**
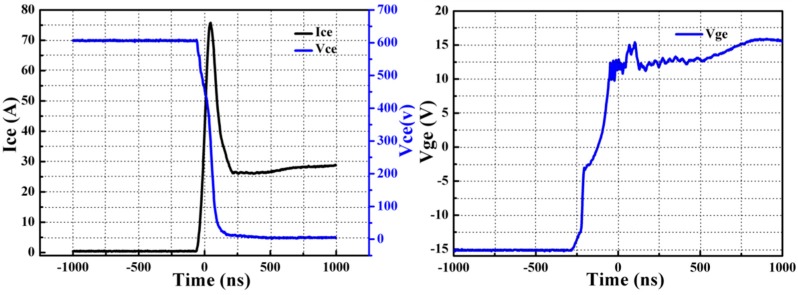
Turn-on behavior of an IGBT at V_DC_ = 600 V, I_C_ = 25 A, and T = 25 °C. (V_ce_ 100 V/div, I_ce_ 20 A/div, V_ge_ 5 V/div, and time 0.25 μs/div.)

**Figure 9:**
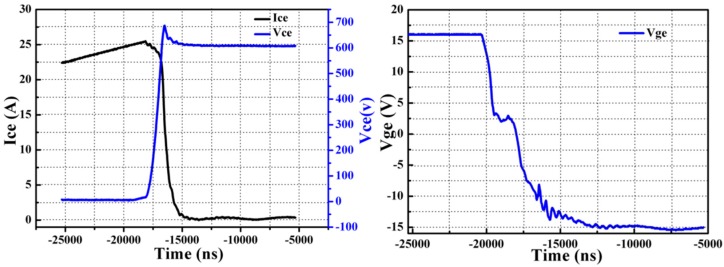
Turn-off behavior of an IGBT at V_DC_ = 600 kV, I_c_ = 25 A, and T = 25 °C. (V_ce_ 100 V/div, I_ce_ 20 A/div, V_ge_ 5 V/div, and time 0.25 μs/div.)

In order to verify feasibility of this pulse-current-assisted-sintering method for bonding IGBTs, it is essential to study static and dynamic characteristics of the IGBT assembly by the pulse-current-assisted-sintering method. The experimental study herein is based on Infineon IGBT chips SIGC32T120R3LE. The chip parameters can be found in details in the datasheet [39]. The switching on and off behavior was characterized by a double-pulse testing method [40]. Both the turn-on and the turn-off gate resistance are 20 Ω. The inductive load during the measurement is set as 200 nH. The collector voltage, gate voltage, and collector current flowing through the IGBTs were measured by an oscilloscope with high-voltage probes. The measured static and dynamic results are compared with the ones of the datasheet, as listed in Table 2. The switching on and off behavior including the collector voltage V_ce_, the collector current I_ce_, and the gate voltage V_ge_ of the IGBT assembly by the rapid sintering of nanosilver paste is presented in Figure 8 (turn-on) and Figure 9 (turn-off).

It is evident that both the measured I_ces_ and V_ce(sat)_ are comparable or even better than the typical values of commercial Device, indicating that the IGBT assembly by the pulse-current-assisted-sintering method is practicable. The IGBTs were not damaged due to such high heating current as others may concern.

The switching on time is defined as the sum of turn-on delay time, t_d(on)_, and rising time, t_r_. The switching off time is defined as the sum of turn-off delay time, t_d(off)_, and falling time, t_f_. The switching on and off time are 76 ns and 450 ns, respectively. This is consistent with the fact that the switching off time is usually at level of several nanoseconds and an order of magnitude higher than the switching on time [41].

Moreover, the total IGBT loss is defined as the sum of IGBT turn-on and turn-off losses. For the switching loss evaluation of the IGBT assembly, a total collector current of 25 A is applied. It is worth noting that the overshoot of the collector voltage is less than 50 V during switching on. However, there is a significant overshoot in the collector current. The maximum collector current during switching off could reach up to ~75 A, which is two times higher than the rated collector current. It was likely that the DBC substrate of the IGBT assembly had been oxidized locally, especially the positions close to the heating electrodes, due to such high heat current. The generated copper oxide should increase the resistance because the electrical resistivity of the copper oxide is much larger than those of both copper and silver. The skin effect of the copper metallization, which could be expressed as δ = [ρ/(π × μ × f)]^1/2^, becomes significant at high frequency. In the above equation, δ is skin depth; ρ is electrical resistivity; μ is permeability; and f is operating frequency. As a result, the skin depth should increase as well as the parasitic inductance once the electrical resistivity increases. Then the variation in parasitic inductance led to the overshoot in the collector current of the IGBT assembly during switching off. The turn-on and turn-off loss are 1.11 mJ and 1.44 mJ, respectively. Unfortunately, there is no typical value of the switching characteristics in the datasheet because switching characteristics is depending strongly on module design and mounting technology and can therefore not be specified for a bare die. Thus, we compared our results with the typical values of a commercial IGBT module (MMG25H120XB6TN, MacMic Co., Ltd., Changzhou, China) [42].

## 4. Discussion

### 4.1. Fracture Surface

**Figure 10:**
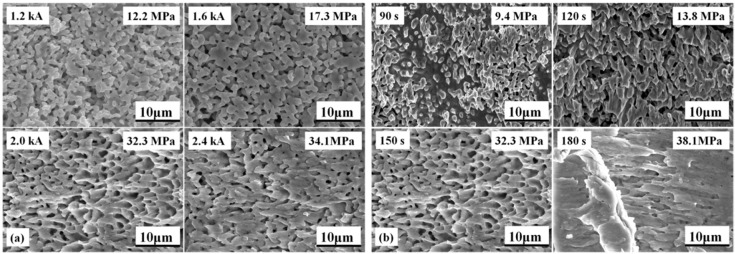
Comparison of microstructures of fracture surface of specimens sintered: (**a**) at different currents (current-on time of 150 s); (**b**) at different current-on times (sintering current of 2.0 kA).

The fracture microstructures of the sintered nanosilver joint at different sintering conditions were investigated by SEM, as shown in Figure 10. Figure 10a shows that the higher the current, the more significant the elongated shape on the fracture surface of the specimen. When the current is less than 2.0 kA, there is no elongated dimple that can be interpreted. It was reported previously that obvious plastic deformation should appear on fracture surface of the sintered nanosilver joint with relatively high shear strength [43]. The fracture failure is a kind of cohesive failure rather than adhesive failure. Then, the shear strength of the sintered nanosilver joint is close to that of soldering joints, e.g., PbSn, AuGe12, and ZnAl5, and pressure assisted sintered joint, i.e., >30 MPa [43,44]. The elongated dimples mean the larger fracture deformation or fracture strain of the sintered nanosilver. If significant elongated dimples are present, the shear strength of the sintered nanosilver could reach at least 30 MPa [23]. The fracture surfaces of the IGBT assemblies that were sintered with different current-on time under the same current of 2.0 kA are shown in Figure 10b. Almost no elongated dimple and plastic flow can be observed in the cases of 90 s and 120 s. Therefore, we considered low shear strength could be used to explain “no elongated dimple”. However, significant plastic deformation can be found in the case of 150 s and 180 s. The shear strength of sintered joints with the current-on time of 150 s and 180 s could, therefore, reach 33.2 MPa and 38.1 MPa, respectively.

### 4.2. Void Distribution

**Figure 11:**
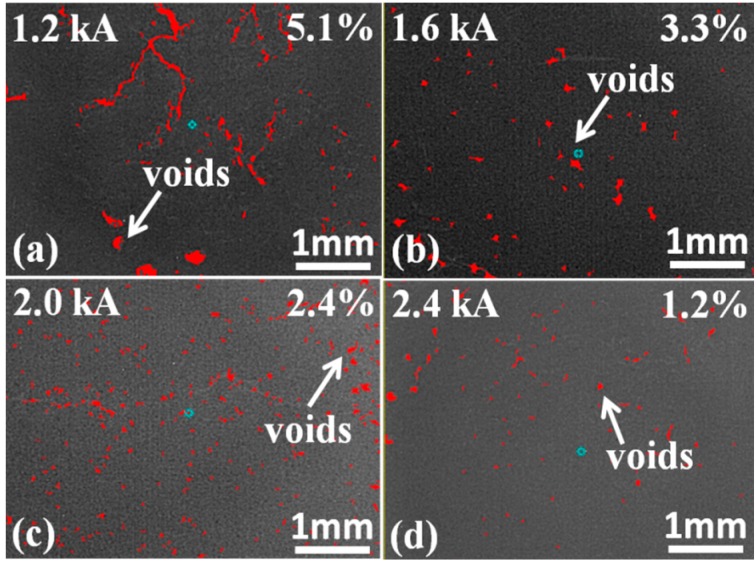
X-ray computed micro-tomography of specimens sintered at: (**a**) 1.2 kA; (**b**) 1.6 kA; (**c**) 2.0 kA; (**d**) 2.4 kA.

Void ratio is also a critical factor that affects die-shear strength and thermal properties of the joints [32]. X-ray micro-tomography (μ-CT), which could identify the void distribution nondestructively based on the criterion of contrast gradient [39], was used to explore the voids in the sintered nanosilver joints of the IGBT assemblies with different sintering current. The right regions as shown in the Figure 11 represent voids or defects in the sintered joints. The area ratio of the right regions could be calculated as the void ratio [33]. More heat could be accumulated at the voids area and then caused the higher junction temperature [34]. During the sintering process initial voids can be transformed into the void with the volatilization of organic matter. Figure 11 shows the microstructures of the sintered nanosilver joint using different sintering current by μ-CT. It can be seen that with an increase of the current, the voids of the joint decreased. Probably because with the temperature increase organic matter has been completely volatile, grain boundary diffusion and lattice diffusion occurred under high temperature to achieve rapid densification of solder paste and the densification process leads to a decrease in the number of voids. Therefore, the density of the sintered joints was enhanced by increasing the current for sintering. It is likely that voids, which could impact the thermal properties of the die attachment greatly [45], are easily formed in the sintered joint and at the interface between the IGBT and the die attachment during the rapid sintering process [36].

### 4.3. Cross Sections

**Figure 12:**
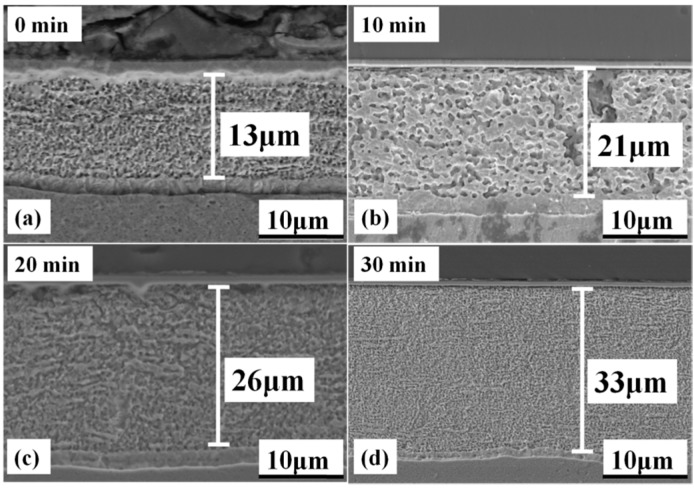
As-sintered bondline thickness of sintered nanosilver with different pre-dried time of: (**a**) 0 min; (**b**) 10 min; (**c**) 20 min; (**d**) 30 min.

Figure 12 shows SEM images of cross sections of the sintered silver joints. The as-sintered bondline thickness of the joint increased when prolonging the pre-dried time at the same sintering current of 2.0 kA. It is proven that the as-dried paste at 90 °C for both 0 min and 10 min is too soft to take the fixing pressure, so that the paste could be squeezed out greatly and the as-sintered bondline thickness reduced to 13 μm and 21 μm non-uniformly, respectively. It is reasonable that the die-shear strength of the joints with the pre-dried time of 0 min and 10 min is less than 10 MPa. Once the pre-drying time was prolonged to more than 20 min, the as-sintered joints have dense and uniform bondlines and the as-dried paste could be squeezed out. The as-printed paste is 90 μm thick compared with the as-sintered bondline of less than 30 μm. The bondline shrinkage was due to the densification of silver nanoparticles. However, pressure-assisted sintered nanosilver is usually shrunk to half of the as-printed bondline thickness [46]. The great shrinkage of the sintering paste using electrical current indicates much higher driving force for the densification of the silver nanoparticles compared with that of conventional hot-pressing sintering of nanosilver paste since the processing time is much shorter this way.

The thermal performance and reliability of die attachment greatly depend on the joint density. Enough current-on time is critical to the formation of good bonds at the interfaces between the sintered silver joint and die/substrate [41]. Furthermore, based on Ivensen’s sintering theory, the densification in the sintering process could be considered as elimination of crystal defects [47]. The longer dwelling time on the peak temperature, which was proven to be only dependent on the sintering current herein, can promote evaporation of organics in the paste, and necking/nucleation of the silver nanoparticles; thereby, the relative density of the sintered silver joints can be enhanced [48]. The conclusion is consistent with the variation of the average thermal resistance of the IGBT assemblies, which decreases as the current-on time increases from 90 s to 180 s. Therefore, it should be preferable to increase the current-on time to maintain the sintering temperature in order to reduce the crystal defects and accelerate the densification of the die-attach layer.

**Figure 13:**
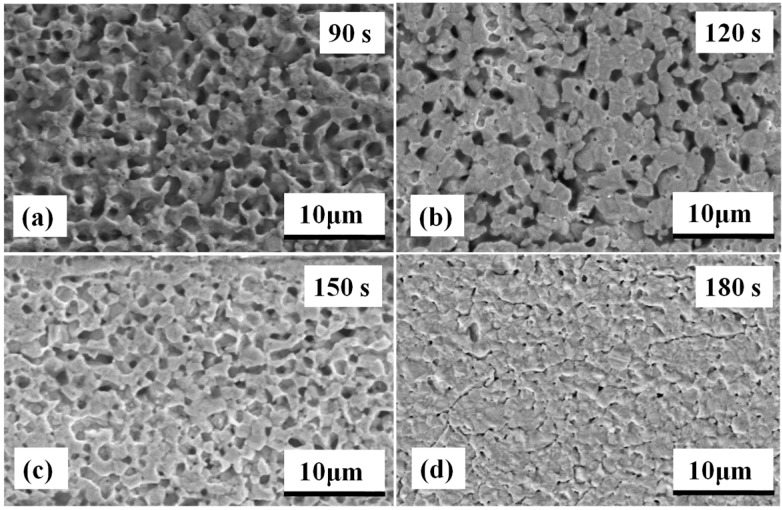
Pore distribution of sintered silver joints sintered at different current-on time of: (**a**) 90 s; (**b**) 120 s; (**c**) 150 s; (**d**) 180 s.

Moreover, the pore size distribution in the sintered silver joints is shown in Figure 13. Figure 13c,d shows that the pores are small and round in shape, which may be beneficial to the thermal resistance of the die attachment [45]. However, larger and more irregular pores emerged in the sintered silver joints, as can be observed in Figure 13a,b. The shorter surface diffusion could hinder further densification and impact the thermal resistance of die attachment ultimately. It is worth noting that the larger and more irregular pores could impact the long-term reliability of the sintered joints because cracks are easily formed in the vicinity of these pores by stress concentration [49]. The current-on time of 150 s is recommended considering both the thermal performance and reliability of the die attachment.

### 4.4. Evolution of Particle/Grain Size

In order to verify that short sintering time could refine grain, we have to deduce the relationship between grain growth and sintering time. The grain boundary migration rate can be expressed as the following equation [50]:

V_b_ = M_b_ × F_b_ (2)

where M_b_ stands for the mobility of grain boundary; and F_b_ is the driving force. The M_b_ is temperature dependent as the following:

M_b_ = (D_b_ × Ω)/(K × T × W_b_) (3)

where D_b_ is the diffusivity of grain boundary; W_b_ is boundary width; Ω is atomic volume; K is Boltzmann constant; and T is absolute temperature. The driving force, F_b_, can be expressed as the following:

F_b_ = A × σ_b_/D_g_ (4)

where σ_b_ is surface energy; and D_g_ is average particle size. Grain boundary diffusion could lead to grain growth, as mention above. Therefore, the grain boundary migration rate is proportional to grain growth rate, i.e., dD_g_/dt.

dD_g_/dt = A × σ_b_ × D_b_ × Ω/(K × T × W_b_ × D_g_) (5)

**Figure 14:**
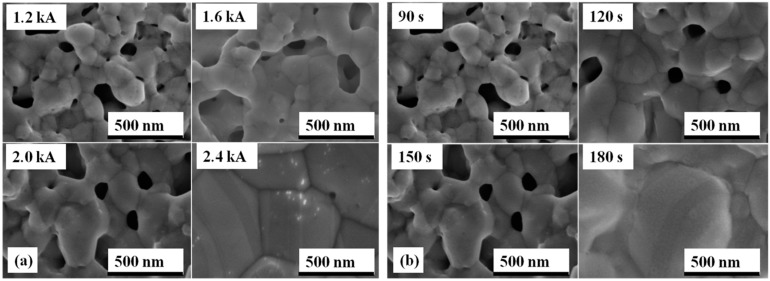
Particle/grain size of nanosilver joints sintered: (**a**) at different currents (current-on time of 150 s); (**b**) different current-on time (sintering current of 2.0 kA).

**Figure 15:**
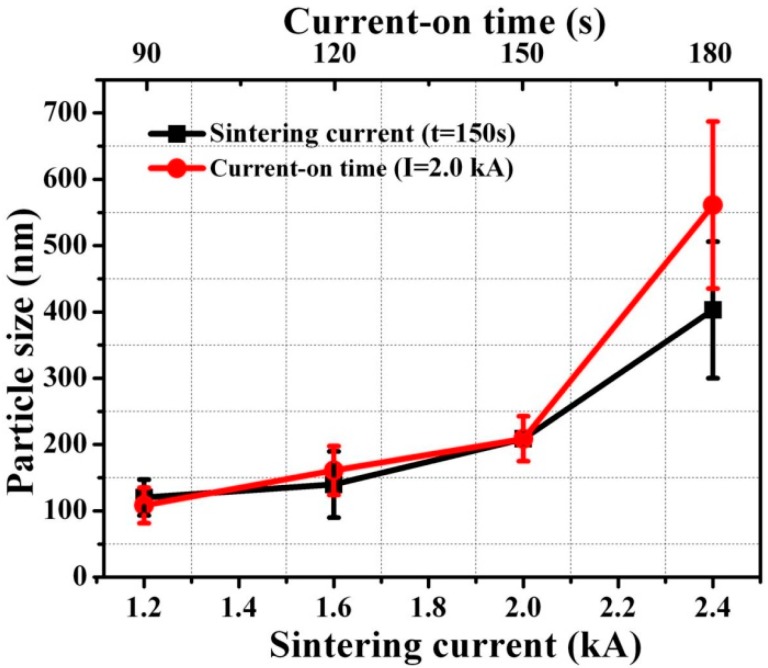
Particle/grain size distribution of nanosilver joints sintered at different currents (t = 150 s) and different current-on times (I = 2.0 kA).

We integrate Equation (4) and attain the following:

D_g_^2^ − D_g0_^2^ = k × t (6)

where D_g0_ is the grain size when t equals zero. These equations are only deduced for analyzing the relationship between grain size and sintering time in a qualitative way, so the constants/parameters in these equations are not determined. As a result, we could conclude that the grain growth should dominate the densification when prolonging the current-on time, especially for 180 s when significant grain coarsening could be observed in Figure 14. Figure 15 shows that the grain size on average increases from ~100 nm to more than 500 nm. The apparent strength and the grain size follow a Hall–Petch [51] type behavior:

σ = σ_0_ + kd^−a^ (7)

where σ is the flow strength; σ_0_ and k are size-independent constants; and a is an exponent typically between 0.5 and 1. In the microscopic view, the increase of grain size could lead to the dissolving of the grain boundary. However, high values for yield stress were considered to be related to the effect of increased grain boundaries, providing additional obstacles for movement of lattice dislocations [51]. Therefore, grain refinement could lead to strength enhancement.

Furthermore, grain refinement of nanocrystals leads to an increase in resistance to failure under stress-controlled fatigue, whereas a deleterious effect was found on the resistance to fatigue crack growth [52]. It is, therefore, not recommended to prolong the current-on time to 180 s. The die-shear strength of the sintered joint under the current of 2.0 kA for 150 s could be as high as 32.3 MPa. One hundred fifty seconds is acceptable as the optimized heating time for the current-assisted sintering of nanosilver paste under the current of 2.0 kA.

## 5. Conclusions

Compared with the traditional hot-pressing way to sinter nanosilver paste, which takes than half an hour, we are able to sinter the paste to bond IGBT chips in a much shorter time, i.e., ~150 s with the help of a pulsed current of ~2.0 kA. We obtained robust sintered joints as die attachment for bonding (1200 V, 25 A) IGBT chips (Infineon, SIGC32T120R3LE) using this rapid method. In this way, the die-shear strength and the thermal resistance of the die attachment could reach up to 30–35 MPa and 0.07 °C/W, respectively. Both the static and dynamic electrical performances are comparable or even partially better than that of commercial IGBT modules, indicating that the IGBT assembled using the pulse-current-assisted-sintering method is practical and will not damage power chips due to such high heating current, as other methods might. This work may guide a rapid process to use nanosilver paste for bonding IGBT chips in the future.

## Figures and Tables

**Table 1:** Summary of sintering conditions using pulsed electrical current.

Condition No.	Current (kA)	Current-on Time (s)	Pre-Drying Temperature (°C)	Pre-Drying Time (min)
1	1.2	150	90	20
2	1.6	150	90	20
3	2.0	150	90	20
4	2.4	150	90	20
5	2.0	90	90	20
6	2.0	120	90	20
7	2.0	180	90	20
8	2.0	150	60	20
9	2.0	150	120	20
10	2.0	150	150	20
11	2.0	150	90	0
12	2.0	150	90	10
13	2.0	150	90	30

**Table 2:** Static and dynamic performance comparison between measured results and values of datasheet.

IGBT (SIGC32T120R3LE) 1200 V/25 A	I_ces_ (uA)	V_ce(sat)_ (V)	t_d(on)_ (ns)	t_r_ (ns)	E_on_ (mJ)	t_d(off)_ (ns)	t_f_ (ns)	E_off_ (mJ)
Measured results	0.02	1.73	62	14	1.11	336	114	1.44
Datasheet	Max. 3.48	Typ. 1.70	Typ. 90	Typ. 30	Typ. 2.4	Typ. 420	Typ. 70	Typ. 1.8
